# Process Evaluation of a Participatory Supportive Return to Work Program for Workers Without a Permanent Employment Contract, Sick-Listed Due to a Common Mental Disorder

**DOI:** 10.1007/s10926-016-9625-6

**Published:** 2016-01-25

**Authors:** Lieke Lammerts, Frederieke G. Schaafsma, Willem van Mechelen, Johannes R. Anema

**Affiliations:** 1 Department of Public and Occupational Health, EMGO + Institute for Health and Care Research, VU University Medical Center, P.O. Box 7057, 1007 MB Amsterdam, The Netherlands; 2 Research Centre for Insurance Medicine, AMC-UMCG-UWV-VUmc, Amsterdam, The Netherlands

**Keywords:** Process evaluation, Return to work, Occupational health care, Worker without employment contract, Randomized controlled trial

## Abstract

*Purpose* This study aimed to perform a process evaluation of a participatory supportive return to work program for workers without a (permanent) employment contract, sick-listed due to a common mental disorder. The program consisted of a participatory approach, integrated care and direct placement in a competitive job. Our main questions were: were these components realized in practice and in accordance with the protocol? The evaluation took place alongside a randomized controlled trial. *Methods* The study population consisted of workers who filed a sickness benefit claim at the Dutch Social Security Agency, professionals of this agency and of vocational rehabilitation agencies. We focused on sick-listed workers and professionals who had actually participated in the intervention. Data was collected mainly by questionnaires. *Results* Only 36 out of 94 intervention group participants started with the program. In half of these cases application of integrated care was reported. Most other steps in the program were completed. However, fidelity to the protocol was low to reasonable. Much delay was observed in the execution of the program and only two sick-listed workers were placed in a competitive job. Still, satisfaction with the participatory approach was good. *Conclusions* Despite the positive evaluation of the participatory approach, the full program was executed less successfully compared to similar programs evaluated in earlier studies. This will probably affect the outcomes of our trial. Findings from this study will help to interpret these outcomes. Nevertheless, more knowledge is needed about experiences of stakeholders who participated in the program. *Trial Registration* NTR3563.

**Electronic supplementary material:**

The online version of this article (doi:10.1007/s10926-016-9625-6) contains supplementary material, which is available to authorized users.

## Introduction

Sick-listed workers without a (permanent) employment contract, such as sick-listed unemployed workers, temporary agency workers and workers with an expired fixed-term employment contract, often face more obstacles for return to work (RTW) compared to sick-listed employees, especially when there is no (longer a) workplace to return to [1, 2]. Mental health problems are frequent reasons for sickness absence within this group [3]. As both the non-permanent employment rate and the absolute number of unemployed workers have increased during the last decade [4, 5], RTW of these workers is a growing concern. With the aim to improve RTW of workers without a (permanent) employment contract who are sick-listed due to a common mental disorder (CMD), we developed the participatory supportive RTW program. We evaluated the cost-effectiveness of this program, compared to usual occupational health care (OHC), in a randomized controlled trial (RCT) [6].

The participatory supportive RTW program is a complex intervention, consisting of various components and involving different stakeholders. The program combines a participatory approach, in which the sick-listed worker is encouraged to develop an action plan for RTW, direct placement in a competitive job and integrated care. In the absence of an employer, the Dutch Social Security Agency (SSA) is responsible for RTW guidance of sick-listed workers who have no (longer an) employment contract. Different OHC professionals of the SSA were involved in the program. Vocational rehabilitation agencies were contracted in order to support the sick-listed workers in searching for a suitable (competitive) workplace.

Because of the complexity of the participatory supportive RTW program, it was important to get insight into the extent to which the program was executed as planned [7]. A process evaluation is a useful method to describe the extent to which components of the intervention are realized in practice [7], to distinguish between components of the intervention [8], to learn about barriers and incentives for future implementation of these components [9], to get insight into perceptions of stakeholders [8] and to assess the quality of the intervention [7]. A process evaluation enables researchers to interpret the results of the (cost-)effectiveness evaluation of an intervention [7, 8, 10]. Moreover, it helps to decide which intervention components should be implemented and which components need some improvement [10]. This is of great importance for people who have to reflect on the (cost-)effectiveness of an intervention, as well as for those who have to decide on implementation of the program in practice.

The aim of the present study was to evaluate the process of the participatory supportive RTW program. Despite the fact that process evaluations of RTW programs have become more common [11–14], this is one of the few studies that investigated the accomplishment of a RTW program in a non-regular work setting, namely in the absence of an employer [13]. Therefore, the present study will contribute to a more comprehensive view on the feasibility of RTW programs.

Our main research questions were: which components of the participatory supportive RTW program were realized in practice and to which extent were these components executed according to the protocol? We also evaluated the procedures used to attract sick-listed workers and professionals for participation in the RCT and their reach, perceived barriers and facilitators for RTW and for implementation of the participatory RTW program and satisfaction of the sick-listed workers and professionals who participated in the program.

## Methods

This process evaluation was conducted alongside a RCT on the (cost-)effectiveness of a participatory supportive RTW program for workers without a (permanent) employment contract who were sick-listed due to a CMD, ‘The Co-WORK’ (in Dutch: ‘SamenWERK’) study. This study was approved by the Medical Ethics Committee of the VU University Medical Center and was registered at the Dutch Trial Register (‘Nederlands Trial Register’) on August 7, 2012 (NTR3563). All participants in the Co-WORK study signed informed consent. The study design has been described in detail elsewhere [6].

Based on the components of a process evaluation defined by Linnan and Steckler [7], we assessed five components: recruitment, reach, dose delivered, dose received and fidelity. In addition, we investigated barriers and facilitators for RTW and for implementation of the program and we evaluated the satisfaction of sick-listed workers and professionals who participated in the program. Below is described how these components were operationalized.

### Study Population

The study population consisted of workers without a (permanent) employment contract who were sick-listed due to a CMD, OHC professionals of the Dutch SSA and case managers of vocational rehabilitation agencies.

#### Sick-Listed Workers

Eligible for participation were unemployed workers, temporary agency workers and workers with an expired fixed-term employment contract, who had applied for a sickness benefit at the Dutch SSA. They had been sick-listed between 2 and 14 weeks, with mental health problems as the main reason for their sickness benefit claim. Sick-listed workers could not participate when one or more of the following exclusion criteria was present: (1) not being able to complete questionnaires written in the Dutch language; (2) a conflict with the SSA regarding a sickness benefit claim or a long-term disability claim; (3) the presence of a legal conflict, e.g. an ongoing injury compensation claim; (4) a sickness absence episode due to a CMD within 1 month before the current sickness benefit claim; (5) already having received usual OHC since the start of the current sickness absence period; (6) pregnancy, up until 3 months after delivery; (7) no signed informed consent form and; (8) no intention to RTW before recovery from symptoms. The latter exclusion criterion was based on findings of two earlier studies, which had revealed that sick-listed workers who believe they should be fully recovered before they RTW, require another RTW intervention [15, 16].

#### OHC Professionals

All participating OHC professionals were working at an SSA front office and participated in the study within an intervention team. These intervention teams consisted of at least one insurance physician, one labor expert and one RTW coordinator. All teams were trained in the participatory supportive RTW program by the researchers. They also received a syllabus with the intervention protocol and practical schemes.

#### Case Managers of Vocational Rehabilitation Agencies

The participating vocational rehabilitation agencies were all certified commercially operating agencies. At each agency one case manager was appointed. These case managers received a detailed instruction for the placement of intervention group participants in a competitive job.

### The Participatory Supportive RTW Program

In the participatory supportive RTW program, the insurance physician, labor expert and RTW coordinator of the SSA together with the case manager of the vocational rehabilitation agency supported the sick-listed worker in the development of a consensus-based RTW action plan and in his or her search for a suitable job. Active participation by the sick-listed worker in the program was stimulated. The labor expert monitored the development of the RTW action plan and was responsible for a safe environment in which the sick-listed worker should feel free to come up with suggestions for achieving RTW. A summary of the consecutive steps of the program is presented in Table 1. The program was based on an existing participatory approach [11] (step 3, 4, and 5). An integrated care approach (step 2) and direct placement in a competitive job (step 6) were added to the initial protocol in order to prevent conflicting advice on RTW by different health care professionals and to create a RTW perspective. A comprehensive description of the program and its development, can be found in the study protocol [6].

**Table 1:** The participatory supportive RTW program

Steps	Explanation
*Step 1. Consult RTW coordinator*	The RTW coordinator examines the sickness benefit claim
	The sick-listed worker receives a take-home-assignment to list and prioritize obstacles for RTW
*Step 2. Consult insurance physician* Within 2 weeks after allocation to the intervention team	The insurance physician performs a medical assessment
	The insurance physician contacts the sick-listed worker’s healthcare provider(s) in order to agree upon RTW options
*Step 3. Inventory of obstacles for RTW*	The labor expert supports the sick-listed worker in identifying and prioritizing obstacles for RTW, from the sick-listed worker’s point of view
	The labor expert supports the RTW coordinator in identifying and prioritizing obstacles for RTW, from a professional point of view
*Step 4. Brainstorm session* Within 2 weeks after meeting the insurance physician	The labor expert summarizes the three main obstacles for RTW identified by the sick-listed worker and the three main obstacles identified by the RTW coordinator
	The sick-listed worker and the RTW coordinator think of solutions to overcome each obstacle for RTW
	The sick-listed worker and the RTW coordinator think of suitable work
	The labor expert tries to reach consensus between the sick-listed worker and the RTW coordinator about solutions and suitable work
	The labor expert summarizes the proposed solutions and suggestions for suitable work in a RTW action plan
*Step 5. Preparation for implementation* Within 1 week after the brainstorm session	The insurance physician considers whether the RTW action plan is in line with the physical and mental work capacities of the participant
	Comments of the insurance physician are integrated into the RTW action plan
	The labor expert sends the final action plan to the sick-listed worker, RTW coordinator and insurance physician
	The labor expert underlines the sick-listed worker’s own responsibility in the search for suitable work
	The labor expert refers the sick-listed worker to a vocational rehabilitation agency for support in the search for a suitable job
*Step 6. Placement in a matching competitive workplace* Within 4 weeks after contracting the vocational rehabilitation agency	The case manager offers the sick-listed worker at least two suitable workplaces
	The sick-listed worker is placed in a suitable workplace
*Step 7. Evaluation* Four weeks after contracting the vocational rehabilitation agency	The RTW coordinator contacts the sick-listed worker and the case manager of the vocational rehabilitation agency to inquire if the sick-listed worker has found/been placed in a suitable workplace
	The sick-listed worker will be supported in the job search by two more vocational rehabilitation agencies, in case the first agency has not been able to place the participant in a suitable job. Support in the job search will be continued for two more months
	The case manager of the vocational rehabilitation agency informs the RTW coordinator on the progress of the job search/placement in a suitable job

### Data Collection

Three months after randomization and allocation to the intervention group, the intervention group participant, the assigned OHC professionals and the case manager of the contracted vocational rehabilitation agency, all received a questionnaire. Participating professionals were asked to indicate which steps of the participatory supportive RTW program had been realized and when. All stakeholders were asked about barriers and facilitators for RTW and for implementation of the program, using a predefined list of possible complicating and facilitating factors, and about their satisfaction with the different components of the program. In addition, participants were asked to evaluate the extent to which they felt that they had been taken seriously by the participating professionals, based on the Patient Satisfaction with Occupational Health Services Questionnaire (PSOHSQ) [17]. Participating professionals were asked to fill out the questionnaire only when the participant had actually started with the participatory supportive RTW program and were asked to inform the researchers when this did not happen.

In addition, written reports were examined, such as the RTW action plans and reports by the vocational rehabilitation agencies. Furthermore, we used data of the baseline questionnaire of the Co-WORK study to give an overview of the characteristics of the intervention group participants at entry into the study [6]. For the evaluation of the recruitment and reach of the Co-WORK study the SSA database was used. In case information was missing, we contacted the responsible participating professional, in order to complete the information.

### Process Measures

#### Recruitment

We defined recruitment as the procedures used to attract sick-listed workers, teams of OHC professionals, and vocational rehabilitation agencies for participation in the Co-WORK study. We described these procedures and illustrated the flow of sick-listed workers in the recruitment process.

#### Reach

At the level of sick-listed workers, reach was defined as the proportion of the target population that had actually participated in the Co-WORK study, including both intervention and control group participants. The target population consisted of all sick-listed workers who had been approached for participation in the study and had been eligible for participation, based on the in- and exclusion criteria. Reach was also investigated at the level of the OHC teams. Information was registered about the front offices of the Dutch SSA that had been approached for participation in the study and the front offices and teams of OHC professionals that actually had participated in the study.

#### Dosage

We combined the dose delivered and the dose received in one evaluation component, the dosage. This component was defined as the extent to which the steps of the participatory supportive RTW program had been completed in practice. We determined for each step in the program in how many cases this step had been completed. Only participants who had actually started with the program were included in these analyses.

#### Fidelity

At a general level, fidelity was defined as the extent to which the participatory supportive RTW program had been implemented according to the protocol. We registered for each participant, which steps of the program had been completed (two points per step). One point was given for fulfillment of the first two steps in the program, as these steps consisted of usual OHC. One point was subtracted in case a step had been completed, but not according to the protocol. By using this scoring system, illustrated in table *S1* (Online Resource 1), it was possible to calculate an overall fidelity score per participant. In case no information was available about the completion of a certain step in the program, no score was given for this step and also no point was subtracted. We defined a score of 0–9 as low fidelity, a score of 9–15 as reasonable fidelity, and a score of 15 as the highest fidelity. A score of 9 could mean that all steps of the program were realized in practice, but not according to the protocol. Therefore, this score was used to differentiate between low and reasonable fidelity. We counted the number of participants in each of the three fidelity categories. In addition, we calculated a mean overall fidelity score, by adding up all overall scores and by dividing this by the number of participants. Only participants who had actually started with the program, were included in these analyses.

To get more insight into the timing of the program in practice, we assessed the duration between the steps of the program in the study and compared this to the maximum duration between these steps according to the protocol.

In addition, we assessed the quality of the three basic intervention components in practice, i.e. integrated care, a participatory approach and direct placement in a competitive job. To assess the quality of the integrated care performed (step 2), we registered the number of cases in which the insurance physicians had contacted the healthcare provider(s) of the participant according to the protocol, which was by telephone.

To assess the quality of the participatory approach (step 3, 4 and 5), we evaluated the content of the written RTW action plans. The International Classification of Functioning, Disability and Health (ICF) was used to classify the identified obstacles for RTW described in the RTW action plans. The ICF is a classification system for (problems in) human functioning [18]. It distinguishes between body functions and structures, activities and participation and between problems that may arise in these three domains of functioning, which are respectively: impairments, activity limitations and participation restrictions. These different domains of human functioning interact with the person’s health condition on the one hand, and environmental and personal factors on the other hand [18]. An obstacle for RTW should either be described as an activity limitation or a participatory restriction, as it has to be clear how the obstacle limits the sick-listed worker to function in work. Subsequently, we registered the number of RTW action plans that contained high quality solutions. In line with Anema et al. [11] the quality of these solutions was assessed by determining whether the solutions were related to the perceived obstacle, a person had been made responsible for fulfillment of this solution, and a timetable for implementation was reported. We also investigated whether the solution had been described clearly, i.e. as a measurable action. Finally, suggestions for suitable work were explored, by investigating the extent to which the RTW action plans contained clear descriptions of suitable work and relevant preconditions for RTW.

The quality of the support by the vocational rehabilitation agencies (step 6) was assessed by determining the mean number of suitable jobs offered to each participant. Moreover, for each participant who had been placed in a workplace, we investigated whether this placement met the prescribed criteria for placement in a suitable competitive job, i.e. an employment contract of at least 3 months resulting in at least 50 percent of the salary of the participant’s last job.

#### Barriers and Facilitators for Realization of RTW and Implementation of the Program

We made an overview of frequently reported barriers and facilitators for realization of RTW. We also described how the investments by the different stakeholders had influenced the execution of the program, according to these stakeholders.

#### Satisfaction and Experiences

For each of the three basic intervention components, the most frequently reported experiences by the different stakeholders were described. In addition, it was investigated how satisfied the participants had been with the guidance of the professionals who had participated in the program.

### Data Analysis

Descriptive statistics (SPSS 22.0 (IBM, 2013) and Excel 2010) were used to analyze the data. For the evaluation of obstacles for RTW, we developed a coding system. Each component of the ICF model was given a different color. These colors were used to code the obstacles for RTW that were written in the RTW action plans. The coding of obstacles was done by the first author and repeated by a research assistant. Disagreements were discussed in order to achieve consensus.

## Results

### Recruitment

#### Sick-Listed Workers

**Fig. 1:**
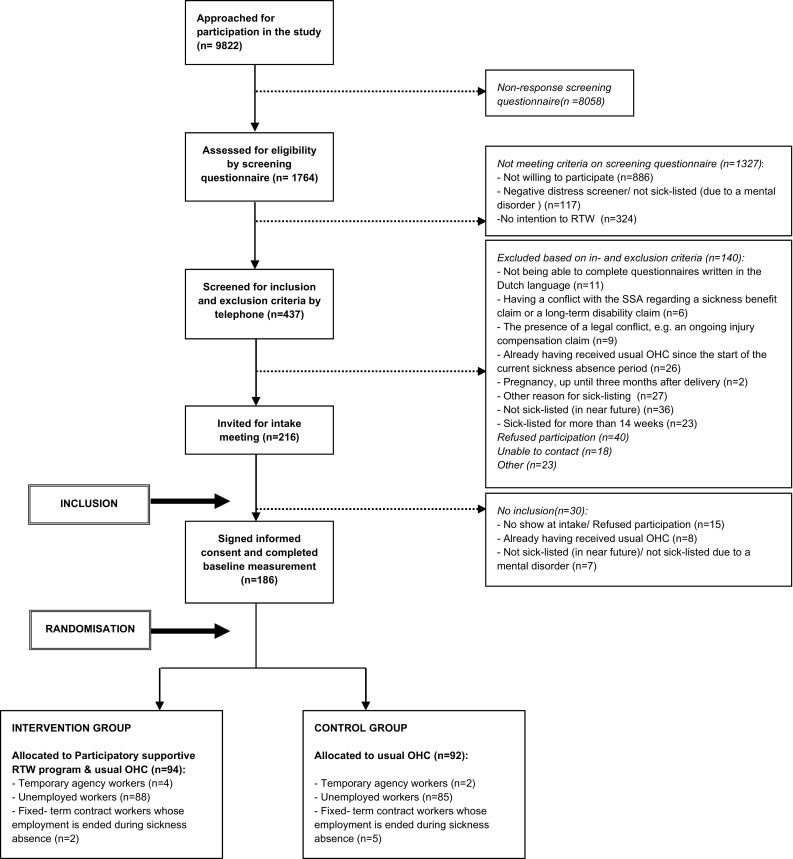
Flow diagram of sick-listed workers in the Co-WORK study

Table 2 presents the recruitment procedures that were used to attract sick-listed workers for participation in the Co-WORK study. The aim was to include a minimum of 168 sick-listed workers in the study. Between March 2013 and September 2014, 9822 sick-listed workers were approached for participation, based on a weekly query of the SSA database. Figure 1 illustrates the flow of sick-listed workers in the Co-WORK study. One important adjustment was made during the recruitment phase. From the end of 2013, the SSA decided to no longer register the reason for sick-listing, in case the sick-listed worker mentioned this reason. From then on, it was no longer possible to recruit participants based on a registered health complaint. Instead, every newly sick-listed worker belonging to one of the participating SSA offices received the invitation package.

**Table 2:** Procedures for recruitment of sick-listed workers in Co-WORK study

Recruitment procedures	Explanation
1. Invitation by Dutch SSA	Workers without a (permanent) employment contract who had applied for a sickness benefit at the SSA because of mental health problems and were belonging to one of the participating SSA offices, received an invitation package from the medical advisor of the SSA 1–2 weeks after sick-listing
	The package included an invitational letter, a flyer with information about the study, a consent form for contact, a screening questionnaire and a return envelope
	The sick-listed workers were invited to fill out the forms, and send these back to the researchers
2. First check of eligibility by screening questionnaire	The returned screening questionnaires were assessed by the researcher or a research assistant for a first check of eligibility
3. Screening for in- and exclusion criteria by telephone	The sick-listed workers with a positive screening result were contacted by the researcher by telephone to give more information about the study and to screen for (other) in- and exclusion criteria
	Sick-listed workers who were screened positive and were willing to participate, were invited to an intake meeting at the SSA
4. Intake meeting at SSA office	Prior to the intake meeting, the sick-listed workers received a brochure with detailed information about the study procedures
	The sick-listed worker was included in the study, after signing informed consent and completion of the baseline questionnaire
	After inclusion, randomization and allocation of the sick-listed worker to the control- or intervention group was performed

#### OHC Professionals

The boards of nine front offices of the Dutch SSA, were approached by the researchers for participation in the Co-WORK study. Each office was asked to form two intervention teams, of which one could serve as a back-up in the situation that the other team was (temporarily) not able to participate in the program. In most cases, the manager invited two existing teams of OHC professionals to participate in the study. In case one of these teams was not willing to participate, another team was approached.

#### Case Managers of Vocational Rehabilitation Agencies

Based on performance indicators, the SSA contracted three commercially operating vocational rehabilitation agencies.

### Reach

#### Sick-Listed Workers

Figure 1 shows that of the 9822 approached sick-listed workers, 619 sick-listed workers were not eligible to participate in the study due to a negative distress screener, an exclusion criterion or for another reason. Of the remaining 9203 sick-listed workers, 186 were included in the study, indicating a reach of 2 %. However, due to a change in recruitment procedures, 7310 sick-listed workers had received an invitation for the study while the SSA had not registered their reason for sickness absence. Many of them would probably not have been eligible to participate, because they were sick-listed for other reasons than mental health problems. An estimation of the actual reach should be based on information about sick-listed workers who had been approached before the recruitment procedure was changed. In total, 2512 sick-listed workers had been approached based on registered mental health problems of which 265 were not eligible to participate in the study. Of the remaining 2247 sick-listed workers, 94 participated in the Co-WORK study (49 intervention and 45 control group participants), resulting in an estimated reach of 4 %.

#### OHC Professionals

Seven out of nine SSA front offices were willing to participate, corresponding to a reach of 78 %. The (perceived) time investment was the main reason for the other offices not to participate. At two offices, only one intervention team was formed. Each team consisted of at least one insurance physician, one labor expert and one RTW coordinator. At the start of Co-WORK, 13 insurance physicians, 12 labor experts and 16 RTW coordinators participated in the study. During the study, one insurance physician, one labor expert and one RTW coordinator were (temporary) replaced by a new professional, because they found a new job/were not willing to participate anymore because of the time investment/were on sickness benefit.

### Dosage

**Fig. 2:**
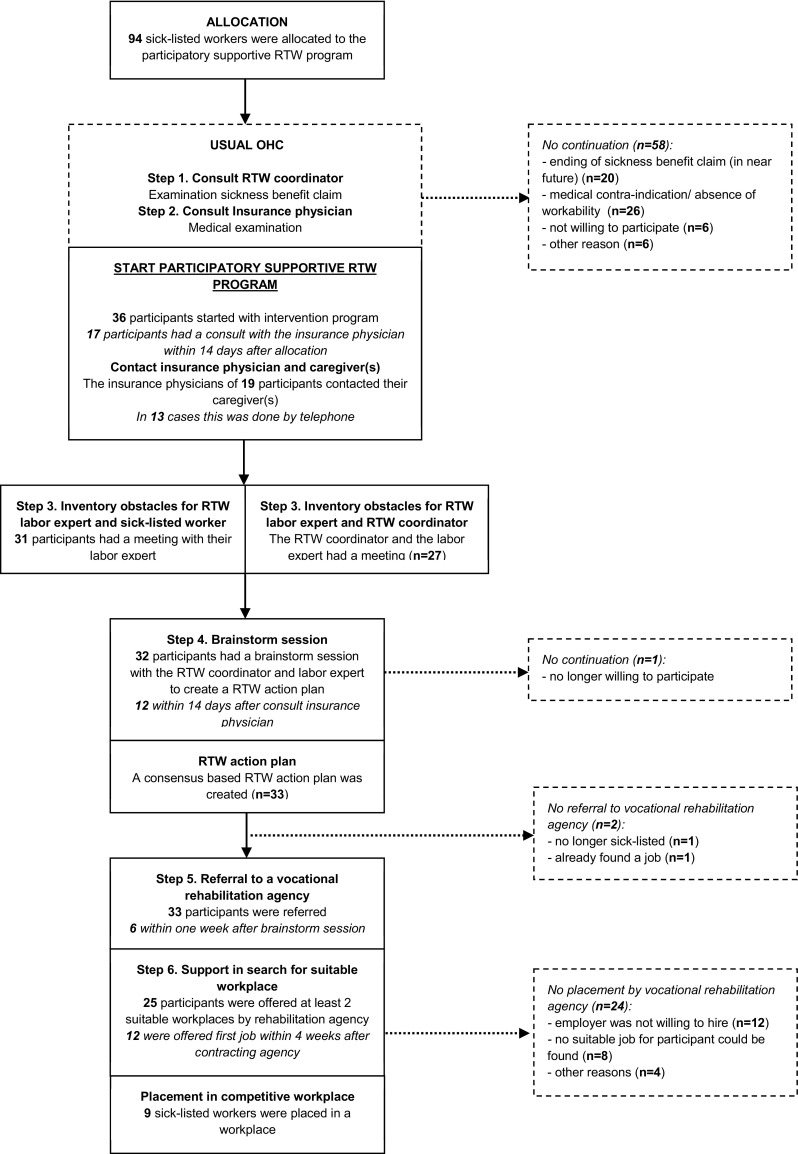
Flow diagram of sick-listed workers in the participatory supportive RTW program

Of the total group of 186 participants in the Co-WORK study, 94 participants had been allocated to the intervention group based on randomization. The flow of sick-listed workers in the participatory supportive RTW program is illustrated in Fig. 2. Of the 94 intervention group participants, 36 participants (38 %) had actually started with the participatory supportive RTW program. Main reasons for not starting with the program were the presence of a (medical) contra-indication and ending of the sickness benefit claim (in the near future). Table 3 describes the baseline characteristics of the participants that started with the program and of the total group of intervention group participants. There were no significant differences between the intervention group participants who had actually participated in the program and those who had not.

**Table 3:** Baseline characteristics

Variable	All intervention group participants (n = 94)^c^	Intervention group participants who actually participated in the intervention (n = 36)
Gender, n (%) female	45 (48 %)	18 (50 %)
Age in years, mean (SD)	45.7 (10.6)	44.3 (9.1)
Type of worker		
N (%) unemployed worker	88 (94 %)	34 (94 %)
N (%) temporary agency worker	4 (4 %)	1 (3 %)
N (%) fixed-term contract worker whose employment ended during sickness absence	2 (2 %)	1 (3 %)
Education^a^		
N (%) low	26 (28 %)	10 (28 %)
N (%) middle	50 (53 %)	20 (56 %)
N (%) high	18 (19 %)	6 (17 %)
Temporary employment contract in last job, n (%)	60 (64 %)	24 (67 %)
Work schedule in last job		
N (%) day work	72 (77 %)	28 (78 %)
N (%) irregular work/flexible schedules	18 (19 %)	7 (19 %)
N (%) shift work	4 (4 %)	1 (3 %)
Working hours per week in last job, mean (SD)	32.6 (11.6)	34.3 (9.0)
Years worked in last job, mean (SD)	10.0 (10.0)	8.3 (9.8)
4DSQ^b^		
Distress scale score, mean (SD)	25.8 (5.1)	25.8 (4.6)
Depressive scale score, mean (SD)	6.6 (3.7)	6.3 (3.3)
Anxiety scale score, mean (SD)	10.7 (6.0)	10.4 (5.8)
Somatic scale score, mean (SD)	14.9 (6.0)	15.7 (6.2)

^a^Low educational level included no education, primary school or lower vocational education; middle educational level included intermediate vocational education or secondary school; high educational level included higher vocational education or university

^b^Range distress scale is 0–32; range depression scale is 0–12; range anxiety scale is 0–24; range somatization scale is 0–32

^c^N varies between 92 and 94 due to missing cases

Most steps of the program were completed in many cases, which corresponds to a high dosage. However, the application of an integrated care approach was reported in slightly more than half of the cases. In some cases, information was missing about the execution of a certain step. Information about the application of integrated care was missing in eight cases (step 2), about the inventory of obstacles for RTW between the labor expert and the participant in three cases and between the labor expert and the RTW coordinator in five cases (step 3), about the brainstorm session in four cases and about the creation of a RTW action plan in two cases (step 4) and about the number of workplaces offered in three cases (step 6).

### Fidelity

#### General Level

In 14 of the 36 cases (39 %) in which the participatory supportive program had been implemented, the fidelity of the application of the program by the intervention providers was low (overall fidelity score 3–9). In the remaining 22 cases (61 %), the fidelity was reasonable (overall fidelity score 9–14). The mean overall fidelity score was 8.9 (SD = 2.2).

Table 4 shows that the mean and median duration between the steps in practice were mostly longer than the prescribed duration by the protocol. In some cases the program was greatly delayed or postponed.

**Table 4:** Timing of the participatory supportive RTW program

Steps	Duration of intervention (in days) according to				
	Protocol (max)	Practice (study)			
		Mean	Median	SD	Range
Allocation to intervention team → Consult insurance physician (n = 35)^a^	14	33.7	15.0	38.7	1–144
Consult insurance physician → Brainstorm session (n = 31)^a^	14	26.0	20.0	21.5	1–80
Brainstorm session → Referral to vocational rehabilitation agency (n = 29)^a^	7	16.7	14.0	13.8	1–62
Referral to vocational rehabilitation agency → First suitable job offered by agency (n = 22)^a^	28	25.6	25.0	18.8	2–84

^a^N differs from number of participants that participated in these steps, due to missing data

#### Integrated Care

In 13 of the 19 cases (68 %) in which the insurance physician reported that he or she had contacted the participant’s healthcare provider(s), the insurance physician had contacted the healthcare provider(s) by telephone.

#### Participatory Approach

Eight out of 33 written RTW action plans (24 %) contained at least one description of an activity limitation or participation restriction, such as the inability to cope with high workload, deadlines or complex issues or a restriction in the available working hours. Most of the RTW action plans (n = 27) contained a description of a personal characteristic, without explaining how this characteristic formed a barrier for RTW. Likewise, in some RTW action plans mental health problems were described, without linking this to RTW. Sometimes only a few words were given instead of a description of an obstacle for RTW. In a few cases a solution was described, instead of an obstacle. The most frequently reported obstacles for RTW were “uncertainty or low self-esteem” (n = 12), “trouble concentrating” (n = 8), “mental health problems” (n = 6), “restriction in available working hours” (n = 3) and “worry” (n = 3).

Almost all RTW action plans (n = 32) contained at least one solution related to the perceived obstacle(s). In all action plans was described who was responsible for the fulfillment of at least one solution. A timetable was present for at least one of the solutions in 28 action plans (85 %). In 25 action plans (76 %), at least one of the solutions was described clearly.

In nine RTW action plans (27 %), both descriptions of suitable work and job examples were given. In 12 RTW action plans (36 %) only descriptions of suitable work were given, such as less demanding work, and in ten action plans (30 %) only examples of a suitable job were listed, e.g. ‘postman’ or ‘mechanic’. In two action plans (6 %) suitable work was not described. Preconditions for work resumption were mentioned in 26 action plans (79 %), e.g. step-wise work resumption and support of a colleague or supervisor at the workplace.

#### Direct Placement in a Competitive Job

On average, each of the participants had been offered three workplaces by the first agency the participant had been referred to. Of the nine workplaces in which participants were placed, only two met the criteria for placement in a suitable workplace.

### Response on Questionnaires for Process Evaluation

Of the 36 participants who had actually started with the participatory supportive RTW program, 31 had filled out the three-month follow-up questionnaire (86 %). A questionnaire had been filled out by the RTW coordinator in 30 out of 36 cases (83 %), by the insurance physicians in 28 cases (78 %), the labor experts in 27 cases (75 %) and the case manager of the vocational rehabilitation agency in 21 cases (58 %). Sometimes questions could not be answered (yet) at the time of the process evaluation, because execution of the program had been delayed or postponed.

### Barriers and Facilitators for RTW And implementation of the Program

The participating professionals often indicated that they did not know whether a certain factor had hampered or facilitated realization of RTW. However, the content of the program was mostly seen as facilitating. To illustrate, in most cases the insurance physician (75 % of the cases), labor expert (93 %), RTW coordinator (63 %) and case manager (57 %), indicated that the development of a RTW action plan had facilitated RTW. Of the participants 55 % indicated that this had facilitated RTW. Also many of them could not tell whether this had been facilitating. This was also true for the job search by the vocational rehabilitation agencies and by themselves.

Many times the insurance physician (43 % of the cases), the labor expert (70 %), the RTW coordinator (57 %) and the case manager (76 %) indicated that their time investment in the program had facilitated a successful execution of the program. This item was also often evaluated as ‘neutral’.

### Satisfaction and Experiences

#### Integrated Care

In more than half of the cases (53 %) in which the insurance physicians reported that they had contacted the participant’s healthcare provider(s), the insurance physicians evaluated the attitude of the healthcare provider as active and cooperative. Often they were also positive about the communication with the healthcare provider(s) (63 % of the cases), and with the degree of agreement that had been reached (53 %). Twenty-one participants reported that they had consulted the insurance physician. Of them about one-third had indicated that their insurance physician was sufficiently aware of the treatment by the general practitioner (GP) or psychologist. Also many of these items were evaluated as ‘neutral’ or ‘not applicable’.

#### Participatory Approach

In many cases the labor expert was positive about the contribution of the participant to the identification of obstacles for RTW (96 % of the cases), the development of solutions to overcome these obstacles (74 %) and the discussion of suitable workplaces (78 %). Often the labor expert also thought that the RTW coordinator had contributed largely to the identification of obstacles for RTW (93 % of the cases), the development of solutions to overcome these obstacles (85 %), and the discussion of suitable workplaces (82 %). Moreover, the labor experts very frequently reported that the participant and the RTW coordinator had reached consensus about solutions (96 % of the cases) and suitable work (93 %). Twenty-three participants indicated that they had visited a labor expert, and the majority (74 %) reported that the labor expert had contributed largely to a sense of security or support and to the perceived equality between the participant and the RTW coordinator (78 %).

#### Direct Placement in a Competitive Job

The case managers of the vocational rehabilitation agencies were more often dissatisfied (24 % of the cases) than satisfied (19 % of the cases) with placement of the sick-listed worker in a suitable job. Also participants were more frequently dissatisfied (36 % of the participants) than satisfied (10 %) with the job offer by the vocational rehabilitation agency. The number of cases in which the RTW coordinator positively evaluated the offering of a suitable job by the agency was equal to the number of cases in which dissatisfaction was expressed (about 30 % of the cases). In the remaining cases these items were evaluated as neutral or not applicable.

#### Satisfaction by Participants

Table *S2* (Online Resource 2) shows how the participants had evaluated the guidance of the OHC professionals who had participated in the participatory supportive RTW program. In table *S3* (Online Resource 3) is presented how the participants generally had appreciated the guidance by all professionals who had participated in the program. Overall, satisfaction was good. However, also many items were evaluated as ‘neutral’ or ‘not applicable’.

## Discussion

The aim of this study was to conduct a process evaluation of a participatory supportive RTW program for workers without a (permanent) employment contract who were sick-listed due to a CMD, alongside the Co-WORK study. The process evaluation revealed that only a small part of all intervention group participants had actually participated in the program. In these cases, the dosage of the program was high. However, the application of an integrated care approach had been reported in only half of the cases. Moreover, fidelity to the program was low to reasonable. This poor fidelity was mainly the result of a delay in the execution of the program and a low number of placements in a suitable competitive job. Nevertheless, most of the stakeholders were satisfied with the use of the participatory approach, which was the core of the participatory supportive RTW program.

### Comparison with Other Studies

Earlier studies have demonstrated good feasibility of similar participatory RTW programs for sick-listed employees with low back pain, employees with distress and sick-listed unemployed and temporary agency workers with musculoskeletal disorders [11–14]. Our process evaluation revealed that the execution of a participatory RTW program aimed at workers without a (permanent) employment contract who were sick-listed due to a CMD was less successful.

Although the program was aimed at a large group of sick-listed workers, in our trial the program seemed to be suitable for only a small group, i.e. those whose sickness benefit was not likely to end in the near future and who had no contra-indication for participation in the program. The percentage of participants with a medical contra-indication in our study (28 %) was much higher compared to the percentage in an earlier study by Van Beurden et al. [13] on a similar participatory RTW program for sick-listed workers with musculoskeletal disorders, which was 13 %. Compared to this study, we also found more delay in the execution of the program [13]. Both studies focused on workers who had filed a sickness benefit claim at the Dutch SSA because they had no employer, but for different health reasons. The high number of medical contra-indications and the delay in the execution of the program are possibly related to the type of health complaints of the sick-listed workers in our study, i.e. mental health problems, and the assessment of these problems by the stakeholders. Another explanation for these differences could be that in the study by Van Beurden et al. [13] the sick-listed workers were placed in a (therapeutic) workplace with ongoing benefits from the SSA, whereas in our study only direct placement in a competitive (paid) job was considered suitable [6].

To our knowledge, this was the first time that direct placement in a competitive job was added to a participatory approach in order to improve RTW of sick-listed workers. Unfortunately, only two sick-listed workers were actually placed in a suitable competitive job by the contracted vocational rehabilitation agencies. Although the support of the vocational rehabilitation agencies was possibly still ongoing at the time of the process evaluation, the number of placements in a competitive job was very low. Moreover, very few sick-listed workers were satisfied with the support by these agencies. This could be a result of a lack of support, but also external factors could have played a role. In the Netherlands, between 2013 and 2014 there was an economic recession, and employment opportunities were limited [5, 19]. This may explain why the case managers of the vocational rehabilitation agencies frequently reported difficulties in finding a suitable job.

Integrated care was another intervention component that was added to the original participatory RTW program. Despite the fact that this was part of the protocol, only in half of the cases the insurance physician reported that he or she had contacted the participants’ healthcare provider(s). This is in line with an earlier study by Anema et al. [20], reporting on the limited communication and collaboration between GP’s and occupational physicians when providing OHC guidance for sick-listed employees.

Compliance to the main intervention component, the participatory approach, was also lower compared to the application of such an approach in earlier studies [11–14]. In many of the action plans, it was not explained how the identified obstacles interfered with RTW. Furthermore, the obstacles for RTW identified in our study mostly expressed feelings of uncertainty and mental health problems, while obstacles identified by sick-listed workers in previous studies were more frequently work-related, e.g. obstacles related to job design and physical or mental workload [11–14]. An explanation for this discrepancy is that in our study almost all sick-listed workers were already unemployed before they became sick-listed.

Despite the often unclear descriptions of obstacles for RTW, most action plans did contain at least one practical solution to overcome these obstacles and clear descriptions or examples of suitable work were given. Moreover, in most cases both the participant and the professionals involved were positive about the way the RTW action plan had been developed, and they all thought this plan would facilitate RTW. The majority of the participants were also satisfied with the coordination by their labor expert, which is in accordance with the high satisfaction with process guidance found in the study of Van Beurden et al. [13]. Possibly, the application of the participatory approach has had the intended function, although the execution of this component in practice—i.e. its form—differed from the protocol. A distinction between form and function of an intervention has been made earlier by Hawe and et al. [21]. They advocated a focus on the function of a complex intervention instead of its form, so that the complexity of this type of interventions could be taken into account [21].

### Strengths and Limitations

An important strength of this process evaluation is that all stakeholders were consulted. This made it possible to integrate experiences of stakeholders with various interests in the OHC field. Consequently, the evaluation of a process evaluation component was seldom based on perceptions of only one stakeholder.

Another strength of our study is that we used a well-known framework to structure our evaluation. The framework of Linnan and Steckler [7] helped us to identify, analyze and describe key process evaluation components.

In this evaluation we distinguished between the three basic components of the participatory supportive RTW program, i.e. integrated care, a participatory approach and direct placement in a competitive job. This enabled us to differentiate between those components of the program that can successfully be implemented in daily practice and those components that still need some improvements. However, by making this distinction we ignored the fact that a complex intervention is more than only a sum of the parts [21]. Also the relations between the intervention components themselves and their relation with the intervention setting, may have affected the execution of the intervention. We did not take these interactions into account, which can be seen as a limitation of our study.

Because of the study design, we were not able to disentangle the reach of the participatory supportive RTW program from the study’s reach, which is a second limitation of our study. Furthermore, it was not possible to determine whether those who did not respond to the invitation for the study would have been eligible to participate, as they were not screened. Possibly, they were not (all) belonging to the target population as was assumed in the calculation of the reach.

Also the recruitment procedures were related to the design of the RCT. Because allocation to the intervention program was based on randomization, it was important that the sick-listed worker was willing to participate in both the intervention and the control group. This process evaluation does not reveal how sick-listed workers can be encouraged to participate in the intervention program.

Another limitation of our study is that mainly questionnaires were used for our data collection. This quantitative research method seemed insufficient to gather data about experiences and satisfaction with the program and about barriers and facilitators for realization of RTW and for implementation of the program in practice. Many of the items to measure these constructs were evaluated as ‘neutral’ or ‘not applicable’.

A last limitation of our study is that probably only sick-listed workers and professionals interested in the Co-WORK study, participated in the participatory supportive RTW program. This may have resulted in selection bias. For this reason, generalizing the results of this study to another context could be difficult.

### Implications for Practice and Research

Despite the positive evaluation of the participatory approach, it is likely that the low compliance measured in this evaluation will affect the outcomes of our trial. The results of this process evaluation will assist us in the interpretation of the effectiveness evaluation of the participatory supportive RTW program. Nevertheless, new research questions have emerged. Further research could investigate the function of the participatory approach according to the stakeholders who participated in the program; perceived barriers for a successful application of integrated care and direct placement in a competitive job; reasons behind the high number of cases in which there was a contra-indication for participation in the program and reasons for delay in the execution of the program. In this way, more in-depth insight will be obtained about the execution of the full program in our trial. This will be helpful in both the interpretation of the trial results and the decision for future implementation of the program. The use of qualitative research methods seem to be most appropriate to address these topics for further research and to unravel processes of implementation and change [22].

## Electronic supplementary material

Below is the link to the electronic supplementary material.

Supplementary material 1 (DOCX 16 kb)

Supplementary material 2 (DOCX 17 kb)

Supplementary material 3 (DOCX 16 kb)
