# Supplementary material for: Process Evaluation of a Participatory Supportive Return to Work Program for Workers Without a Permanent Employment Contract, Sick-Listed Due to a Common Mental Disorder
Source: J Occup Rehabil. 2016 Jan 25;27(2):159–72. doi: 10.1007/s10926-016-9625-6 (PMC5405103; doi:10.1007/s10926-016-9625-6)
Supplement: Supplementary file 1 — Supplementary material 1 (DOCX 16 kb) [file 10926_2016_9625_MOESM1_ESM.docx]

*Supplementary material to article by L. Lammerts*^1^*, F.G. Schaafsma, W. van Mechelen and J.R. Anema ‘Process evaluation of a participatory supportive return to work program for workers without a permanent employment contract, sick-listed due to a common mental disorder’ in Journal of Occupational Rehabilitation*

^1^Department of Public and Occupational Health, EMGO+ Institute for Health and Care Research, VU University Medical Center. E-mail: [l.lammerts@vumc.nl](mailto:l.lammerts@vumc.nl)

**Table S1. Fidelity scoring system**

| Steps | Fidelity score |
| --- | --- |
| *Step 1 and 2: consult with RTW coordinator & consult insurance physician (usual care)* | 1 |
| *Consult insurance physician took place more than two weeks after allocation to intervention team* | - 1 |
| *Insurance physician contacted healthcare provider(s) of participant* | 2 |
| *Insurance physician contacted healthcare provider(s) not by telephone* | - 1 |
| *Step 3: inventory of obstacles for RTW* | 2 |
| *Only the participant and the labor expert had a meeting* | - 1 |
| *Only the RTW coordinator and the labor expert had a meeting* | - 1 |
| *Step 4: brainstorm session* | 2 |
| *Brainstorm session took place more than two weeks after meeting insurance physician* | - 1 |
| *Action plan for RTW was written* | 2 |
| *Step 5: Sick-listed worker was referred to vocational rehabilitation agency* | 2 |
| *Contracting agency took place more than one week after brainstorm session* | - 1 |
| *Step 6: vocational rehabilitation agency offered two suitable jobs* | 2 |
| *First job offer was more than four weeks after contracting agency* | - 1 |
| *Placement in a suitable job by vocational rehabilitation agency* | 2 |
| Maximum total score: | 15 |
