# Supplementary material for: Process Evaluation of a Participatory Supportive Return to Work Program for Workers Without a Permanent Employment Contract, Sick-Listed Due to a Common Mental Disorder
Source: J Occup Rehabil. 2016 Jan 25;27(2):159–72. doi: 10.1007/s10926-016-9625-6 (PMC5405103; doi:10.1007/s10926-016-9625-6)
Supplement: Supplementary file 2 — Supplementary material 2 (DOCX 17 kb) [file 10926_2016_9625_MOESM2_ESM.docx]

*Supplementary material to article by L. Lammerts*^1^*, F.G. Schaafsma, W. van Mechelen and J.R. Anema ‘Process evaluation of a participatory supportive return to work program for workers without a permanent employment contract, sick-listed due to a common mental disorder’ in Journal of Occupational Rehabilitation*

^1^Department of Public and Occupational Health, EMGO+ Institute for Health and Care Research, VU University Medical Center. E-mail: [l.lammerts@vumc.nl](mailto:l.lammerts@vumc.nl)

**Table S2. Participants’ evaluation of guidance OHC professionals participating in intervention**

| N=31 | | |
| --- | --- | --- |
| *Since you became sick-listed, have you seen the insurance physician of the SSA?* | *yes:* | *21 (68%)* |
| The insurance physician… | (totally) agree: | |
| properly understood my health problems |  | 19 (91%) |
| properly understood my problems with work resumption |  | 17 (81%) |
| treated me nicely |  | 19 (91%) |
| knew what he/she was talking about |  | 19 (91%) |
| gave me good advice about my health |  | 16 (76%) |
| treated my complaints confidentially |  | 17 (81%) |
| *Since you became sick-listed, have you seen the labor expert of the SSA?* | *yes:* | *23 (74%)* |
| The labor expert… | (totally) agree: | |
| properly understood my problems |  | 20 (87%) |
| treated me nicely |  | 23 (100%) |
| knew what he/she was talking about |  | 23 (100%) |
| gave me good advice about my RTW possibilities |  | 16 (70%) |
| seemed knowledgeable |  | 22 (96%) |
| *Since you became sick-listed, have you seen the RTW coordinator of the SSA?* | *yes:* | *23 (74%)* |
| The RTW coordinator… | (totally) agree: | |
| properly understood my problems |  | 17 (74%) |
| treated me nicely |  | 20 (87%) |
| knew what he/she was talking about |  | 20 (87%) |
| gave me good advice about my RTW possibilities |  | 12 (52%) |
| seemed knowledgeable |  | 18 (78%) |
