# Supplementary material for: Process Evaluation of a Participatory Supportive Return to Work Program for Workers Without a Permanent Employment Contract, Sick-Listed Due to a Common Mental Disorder
Source: J Occup Rehabil. 2016 Jan 25;27(2):159–72. doi: 10.1007/s10926-016-9625-6 (PMC5405103; doi:10.1007/s10926-016-9625-6)
Supplement: Supplementary file 3 — Supplementary material 3 (DOCX 16 kb) [file 10926_2016_9625_MOESM3_ESM.docx]

*Supplementary material to article by L. Lammerts*^1^*, F.G. Schaafsma, W. van Mechelen and J.R. Anema ‘Process evaluation of a participatory supportive return to work program for workers without a permanent employment contract, sick-listed due to a common mental disorder’ in Journal of Occupational Rehabilitation*

^1^Department of Public and Occupational Health, EMGO+ Institute for Health and Care Research, VU University Medical Center. E-mail: [l.lammerts@vumc.nl](mailto:l.lammerts@vumc.nl)

**Table S3. Participants’ general satisfaction with guidance participating professionals in intervention**

|  |  | N=31 |
| --- | --- | --- |
| To what extent are you satisfied with the guidance by the insurance physician | (Very) satisfied | 16 (52%) |
|  | (Very) dissatisfied | 5 (16%) |
|  | Neutral/ N.A. | 10 (32%) |
| To what extent are you satisfied with the guidance by the labor expert | (Very) satisfied | 17(55%) |
|  | (Very) dissatisfied | 3 (10%) |
|  | Neutral/ N.A. | 11 (36%) |
| To what extent are you satisfied with the guidance by the RTW coordinator | (Very) satisfied | 15 (48%) |
|  | (Very) dissatisfied | 5 (16%) |
|  | Neutral/ N.A. | 11 (36%) |
| To what extent are you satisfied with the guidance by the case manager of the vocational rehabilitation agency | (Very) satisfied | 10 (32%) |
|  | (Very) dissatisfied | 5 (16%) |
|  | Neutral/ N.A. | 16 (52%) |
